# Multi-level barriers & priorities accorded by policy makers for Neonatal Hearing Screening (NHS) in Pakistan: A thematic analysis

**DOI:** 10.12669/pjms.35.6.703

**Published:** 2019

**Authors:** Nazia Mumtaz, Muhammad Naveed Babur, Ghulam Saqulain

**Affiliations:** 1 Dr. Nazia Mumtaz, PhD (Rehabilitation Sciences), In Charge Post Graduate Programs, Allied Health Sciences, Shifa Tameer-e-Millat University, Islamabad, Pakistan; 2 Dr. Muhammad Naveed Babur, PhD (Rehabilitation Sciences), Dean, Isra Institute of Rehabilitation Sciences, Isra University, Islamabad, Pakistan; 3 Dr. Ghulam Saqulain, F.C.P.S (Otorhinolaryngology), Head of Otorhinolaryngology Department, Capital Hospital, Islamabad, Pakistan

**Keywords:** Hearing loss, Neonatal Screening, Policy, Quality of life, Language development

## Abstract

**Objectives:**

To explore the Barriers and Priorities accorded to neo-natal hearing screening at government health policy level.

**Methods:**

This exploratory descriptive study design employed qualitative parameters with purposive sampling and was conducted at Capital Administration & Development Division (CADD) and Ministry of National Health Services, Regulations & Coordination (MoNHSR&C) from June 2015 to January 2017 over a period of 18 months. Sample included stakeholders concerned with healthcare planning and policy making in Pakistan. Research included in depth interviews using a self-structured interview guide from three policy makers’ fulfilling the selection criteria. Data recorded was transcribed and thematic analyses drawn manually and verified using two separate coders.

**Results:**

Outcomes from thematic analysis drawn were Planning, Funding, Governance, Awareness, Medical and technical capacity building, Policy development, Evaluation and Sustainability. Lack of financial resources allocation due to policies and legislation top the list of barriers. The lack of research and reliable data as to the number of persons suffering from hearing loss (HL) from birth and its economic cost to the state and individual remains a policy barrier.

**Conclusion:**

The barriers to NHS are lack of financial resource allocation by the federal government, and lack of research and reliable statistics about Hearing Impairment (HI) and its economic cost.

## INTRODUCTION

Pakistan is a developing country ranked as the sixth populous in the world^1^ with a significant part of its population suffering from hearing impairment (HI) beset with late identification of hearing loss (HL) more often seen in the age group of 19-24 months i.e., 48% compared to 22% at 0-6 months.^2^ This is compounded by a fragile health care system which is not responsive enough to address the health problems of the people let alone initiate curative or management programs for hearing impaired children. The same has been pointed out by Tucci D et al. in their study and concluded that “high prevalence of HL in developing world was due to a variety of factors, including lack of widespread comprehensive immunization programs and medical care, and inadequate funds for intervention once HL is identified”.^3^

Neonatal hearing screening (NHS) is a public health care initiative to identify and detect infants born with impaired hearing from birth to one month of age to identify those infants with hearing loss. The purpose of NHS of newborns is to detect infants having hearing loss (HL) so as to appreciably bring down the age of identification of HL in children for intervention to take place by six months of age in order to provide better speech language development, education and quality of life (Qol).^4^ NHS is well established in developed countries. However, different barriers have played negative role to stop the introduction and success of such programs in low and middle income countries.^5^

Though parents of hearing impaired children in Pakistan feel that detection of HL in their children at an early age was beneficial^6^, however, there was no NHS program in the country worth mentioning.^7^ Also data on Pakistan with the World Bank (WB) shows that US$ 37 per capita is being spent on health as against the recommended US$ 44 per capita as per WHO guidelines^8^, both show a dismal position.

In Pakistan keeping in view late identification of HL in children, lack of resource allocation to such issues, cultural constraints, inadequate trained health personnel, it seems Pakistan faces multiple level barriers in initiating NHS programs. Therefore, this study was conducted to identify the barriers and the priorities accorded to neonatal hearing screening by policy makers in Pakistan.

## METHODS

This exploratory descriptive study, employing purposive sampling technique and qualitative parameters was conducted in Islamabad over a period of 18 months from June 2015 to January 2017, following approval of institutional research ethics committee.(1402-PhD-011 October 2, 2015) Sample included stake holders directly concerned with planning and policy making in the healthcare system of Pakistan, having knowledge of prevalent situation. This was done as neonatal hearing screening is required at national level and for this policy needs to be formulated for enactment of legislation. Stake holders of disability policy and others were excluded from the study since disability policy concerns with children in later years, when hearing and speech disability has manifested. The research was conducted with in depth interviews in English, using interview guide. Five policy makers were approached using personal contacts and interviews were conducted from three policy makers from Capital Administration & Development Division (CADD) and Ministry of National Health Services, Regulations & Coordination (NHSR&C) who consented for inclusion in the study. Interview guide was developed and tested with a policy maker bureaucrat (civil servant) to determine how discussion was led by the formulated questions in the interview guide. It contained easily comprehendible questions with the flow modified and rearranged in a sequence including policy cover, legislative cover, NHS in Pakistan, fiscal resources, linkage between external donor agencies and health care programs and awareness through health and social network. Further, the probes of each domain were built in to the relevant sections of the interview guide, which served to steer the discussion into the domain areas without leading the respondents. The Principal Investigator (PI) provided just enough direction for the respondents to focus on the domains for exploration of multi-level barriers with reference to NHS, and allowed the respondents to express their views at length and in an uninhibited manner. Procedure included proper appointment, in depth and in person interviews which were conducted in their respective offices at CADD and NHSR&C, digital recording of the interview, with consent of the participant. Questions about wide concepts of barriers were put forward to extract extensive exhaustive answers and probes were resorted to when extensive information was sought. Though interview guides were used, participants were allowed to respond freely and response time varied among the participants ranging from one and a half hour to three hours.

Data was subjected to content analysis regarding NHS in Pakistan and audio-recordings were transcribed and thematic analyses were drawn manually and verified with the help of two separate coders including a Pl and a healthcare researcher. Initially broad codes were derived from the areas of exploration of barriers to NHS at policy level, within which the views & first hand experiences were organized. Initial coding allowed pattern recognition and emergence of the initial themes. The two coders independently came up with themes and sub-themes within the transcripts. The differences in coding were exhaustively discussed and were resolved by refining the definitions of codes, creating new codes or collapsing low level codes. Thus a repetitive pattern was used and the coding process followed iterative pattern with modifications of codes to the satisfaction of the researcher and application of codes to the data. Any new themes emerging from the data that did not fit into any of the agreed upon codes were assigned new codes and included in the analysis. The fine-tuning and emergence of results was so designed to lead towards the forming of rational and consistent arrangements.

## RESULTS

The thematic analysis of data collected during in depth interviews with policy makers revealed the themes that emerged within each domain of NHS and the barriers to NHS. The outcomes drawn from the thematic analysis were Planning, Funding, Governing, Awareness, Capacity building (medical and technical), Policy development including Legislation, Administration, Evaluation and Sustainability. The themes and linkages of these themes to the emerging characteristics in the interviews are mentioned in Table-I.

**Table I:** Thematic Analysis: Themes and Emerging Characteristics.

S. No.	Themes	Characteristics
1	Planning	Priority, identification and highlighting of HI as disease, visionary ideas, sustainability of NHS programs and maturity of health care system, acknowledging the rights of the child, legislative action in public health policy. Federal and provincial health polices
2	Funding	Access to international funding and prioritization in national financial allocation
3	Governance	Dedicated leadership, support from international & national donor agencies, establishment of NHS, leveraging government resources, leveraging resources from NGOs.
4	Awareness	Advocacy and public awareness, Media awareness and focus on HI as significant public health matter, education of policy makers, professionals, the public and community, addressing stake holders using top to bottom approach in health care system.
5	Medical & technical capacity Support	Availability and capacity building of professional manpower. Acceptability of HI management through early intervention in medical community Technical training and knowledge sharing, Accuracy and availability of equipment
6	Policy development and legislation	Developing protocols and policies at the level of GOP, WHO, Institutionalized policies and protocols at the level of Tehsil Head Quarter Hospital (THQH), and evaluation thereof, universal accessibility as a right. Legislation of NHS at federal and provincial level
7	Administration	Focused leadership, Planning and policy development and implementation, ensure optimized medical management in HI
8	Evaluation	Outcome monitoring, indicators of successful functioning, quality assurance program based on digital records.
9	Sustainability	System integrated as a functioning public health system and adequately financed, Part of national health program, Program administration, equipment, education, public relations and follow-up, evaluation of outcomes

**Table II:** Thematic Analysis: Barriers to NHS at policy level.

S. No.	BARRIERS
1	Lack of policies, legislation and fragile health system at federal & provincial level
2	Gap between federal and provincial health policies
3	Lack of scientific focus during policy formation
4	Lack of contact between primary specialty physician and new born
5	Lack of neonatal screening tools, infra-structure, equipment and fiscal resources
6	Lack of advocacy and public awareness
7	Not being a sensational issue hence not focus of electronic and print media
8	Lack of services and trained human resource
9	Socioeconomic cultural constraints
10	Deficiencies of community health care system
11	Lack of primary prevention at THQH
12	Lack of technical advice by WHO and international donor agencies
13	Lack of evidence based surveys to identify the magnitude of HI and prioritization of NHS

## DISCUSSION

Thematic analysis conducted to explore the Barriers and Priorities accorded to neo-natal hearing screening at government health policy level in the current study was consistent with other studies wherein it was universally accepted that newborns should be screened for HI by one month of age, diagnosed by three months of age, and intervened no later than six months of age^9^,^10^ for enabling a HI child to compete with his peers in the academic and social fields.

In the current study, targeting the health policy makers, they opined that in Pakistan on account of non-availability of funds for NHS complemented by subsequent remedial and rehabilitation program, NHS programs are unlikely to see the light of the day. This is also indicated in other studies that this state of affairs in developing countries is the leading cause of delay in diagnosis, competing diseases, cultural and social norms, with scarcity of funds for NHS being the major obstacle in launching preventive and therapeutic steps resulting in higher prevalence of HL.^3^

The findings of the present study indicate that the first prerequisites for NHS programs is policy cover followed by legislative cover as well as adequate and credible statistics and facts to emphasize that HI was a disease with a severe economic impact on the state. It was stressed repeatedly by the health policy makers that in order to market NHS the media awareness needs to be created which is only possible through sensationalizing the negative impact of HI on society on the pattern of how external donor agencies were sponsoring campaigns for communicable and non-communicable diseases like Dengue, Polio and Hepatitis and AIDs. In an Indian study by Baxipatra D, one barrier was identified as disability insensitive attitude of the society and therefore, Laws and regulations however stringent can accomplish little unless there is a perceptible shift in the outlook of the community to the HI population unless the media through its power of sensationalism and glamour creates a conducive atmosphere for NHS legislation by positively influencing policy makers or legislators.^11^

In this study the policy makers expressed concerns that lack of policies is attributable to sheer default by the health authorities and legislators of Pakistan. Invoking international obligations and covenants such as the UN Declaration of the Rights of the Child can persuade government policymakers of their responsibilities in providing NHS as a preventative measure. Getting the right degree of recognition at the policy level involves political cognizance and resolution.^12^ It was maintained by the policy makers that gaps exist in critical areas of legislation at the federal and provincial government level manifested in the fragile health care system in the country. The present study indicates that barriers include the lack of support in rural areas, finances of the parents, and cultural and linguistic obstacles. Also, Olusanya BO et al. has reported in a related study that restrictions of funds, manpower scarcity, lack of support services, absence of public cognizance and ambiguity concerning the pledge from healthcare professionals are challenges encountered but are not unbeatable.^13^ As HI is an invisible disability not sensational in nature therefore it could not become the focus of press and electronic media.^10^ Policy makers found support in the postulate for audiological services and referrals to be combined with an effective follow up system, based upon digital record to ensure a sustainable NHS with positive outcomes.^12^

It was enunciated by the health policy makers that public health spending provides an insight into a country’s health progress as reflected by World Bank data illustrating that Pakistan spends just US$ 37 per capita on health, much lower than WHO’s prescribed level, essential for vital health needs, with only 0.42 % of GDP is allocated to health in Pakistan.^8^ The policy makers stated in the present study that costs involved are extremely high for NHS in view of the high birth rate and scarcity of fiscal resources. Tucci D et al. also noted in their study that inadequacy of funds was an important factor.^3^

The findings of this study indicate that the Pakistan’s Ministry of NHSR&C, administers public health policy in different manners, including laws, policies, administrative orders, and policy-related rules and regulations with a degree of flexibility. In contrast, a developing program in China provides examples of how legislative language has been used. In China, Presidential Order No 33, Article 24 (1994) states that “… medical and health institutions shall gradually develop medical and healthcare services such as the screening of newborn babies.”.^12^ Unrealistic expectations persist in the minds of policy makers from international agencies such as WHO for extending technical advice for NHS for HI as the international health and donor agencies have their own agendas to adhere to with their source of funding being from those countries whose interests are first and foremost of their concern^14^, therefore our national health policies are influenced by priorities of international health organizations.

In was highlighted by health policy makers that it is appropriate to adopt successful models of NHS.^15^,^16^ The absence of UNHS in Pakistan can partially be attributed to lack of interest on the part of the policy makers leading to non-enactment of legislation in this particular field. Highlighting cases as of one HI child detected from NHS followed by early intervention with its consequential benefits is the best sales pitch for NHS for health policy makers.^17^

Except for the province of Sindh, the federal governments and the other three provinces have not legislated on NHS. In the USA as of 2012, 44 states have passed legislation related to NHS.^18^ A lack of integrated, holistic, national based approach to NHS coupled with the limited outreach of the healthcare structure and low incidence of hospital births aggravates the fragile health care system.

### Limitation of the study

It includes approaching and getting time from the policy makers, since they were holding important positions in sitting government and convincing them for recording and presence of a note taker prior to consent. Also slight bias was noted towards neonatal screening system and the health policy makers went to the extent of labeling HI and NHS as not being an issue of public health care importance. Yet they could not elaborate any further as to why disability which is proven to affect many citizens of the country and costs dearly to the state in economic terms, is internationally recognized as a significant health issue with NHS being implemented in the developed and developing countries.^19^ Several studies have suggested that HI persons are entitled for screening against communicable and non-communicable diseases both being faced with social, economic and earning capacity discrimination placing them at an disadvantageous position with the root cause lying in late detection of HI.^20^ To retain national uniformity NHS programs need to be based on a national approach.^12^,^21^

## CONCLUSION

Lack of awareness about the lifelong social and economic impact of HI to an individual remains a formidable policy level barrier to the introduction of NHS in Pakistan and loom in the shape of dire lack of financial resources or allocation by the federal government towards the health sector impacting upon initiation of NHS programs. Moreover, lack of availability of updated and reliable data as to the number of persons suffering from HI from birth is a challenging policy level barrier as is also the dearth of research on prevalence and economic fallout of HI. The policy makers are not sensitized to the inherent costs borne by a HI person from birth and incurred in case of any subsidies extended by the state. One evident barrier to priority accorded to NHS at governmental health policy level, is that HI unfortunately remains an invisible disability, requiring a media blitz and the ensuing melee of fringe benefits accruing from the international health donor organizations.

### Authors’ Contribution:

**NM:** Conceptualization of work, designing of research, methodology & literature review, is responsible for integrity of research.

**NB:** Data analysis and interpretation.

**GS:** Critical revision of the article.
